# Fabrication of Novel n-n Heterojunction Bi_2_O_2_CO_3_/AgVO_3_ Photocatalytic Materials with Visible-Light-Driven Photocatalytic Activity Enhancement

**DOI:** 10.3390/ma18204705

**Published:** 2025-10-14

**Authors:** Weijie Hua, Huixin Yuan, Songhua Huang

**Affiliations:** School of Intelligent Equipment Engineering, Wuxi Taihu University, Wuxi 214064, China; yuanhuixin2000@126.com (H.Y.); songhua.huang@wxu.edu.cn (S.H.)

**Keywords:** photocatalysis, Bi_2_O_2_CO_3_/AgVO_3_, visible light, heterojunction, dye degradation

## Abstract

This research successfully synthesized a novel n-n heterojunction Bi_2_O_2_CO_3_/AgVO_3_ nanocomposite photocatalyst via the in situ chemical deposition process. Characterization results strongly confirmed the formation of a tight heterojunction at the Bi_2_O_2_CO_3_/AgVO_3_ interface. The nanocomposite exhibited characteristic XRD peaks and FT-IR vibrational modes of both Bi_2_O_2_CO_3_ and AgVO_3_ simultaneously. Electron microscopy images revealed AgVO_3_ nanorods tightly and uniformly loaded onto the surface of Bi_2_O_2_CO_3_ nanosheets. Compared to the single-component Bi_2_O_2_CO_3_, the composite photocatalyst exhibited a red shift in its optical absorption edge to the visible region (515 nm) and a decrease in bandgap energy to 2.382 eV. Photoluminescence (PL) spectra demonstrated the lowest fluorescence intensity for the nanocomposite, indicating that the recombination of photogenerated electron–hole pairs was suppressed. After 90 min of visible-light irradiation, the degradation efficiency of Bi_2_O_2_CO_3_/AgVO_3_ toward methylene blue (MB) reached up to 99.55%, with photodegradation rates 2.51 and 2.79 times higher than those of Bi_2_O_2_CO_3_ and AgVO_3_, respectively. Furthermore, the nanocomposite exhibited excellent cycling stability and reusability. MB degradation was gradually enhanced with increasing the photocatalyst dosage and decreasing initial MB concentration. Radical trapping experiments and absorption spectroscopy of the MB solution revealed that reactive species h^+^ and ·O_2_^−^ could destroy and decompose the chromophore groups of MB molecules effectively. The possible mechanism for enhancing photocatalytic performance was suggested, elucidating the crucial roles of charge carrier transfer and active species generation.

## 1. Introduction

With the fast growth of textile consumption and industrialization, dye-containing wastewater has become one of the most severe sources of global water pollution [1]. Primary sources of dye wastewater include dye and auxiliary chemical manufacturers, textile pretreatment, dyeing, printing, and finishing processes, leather processing, papermaking, and printing [2]. According to authoritative reviews, wastewater generated from textile dyeing, finishing, and related processes accounts for approximately 17–20% of global industrial water pollution [3]. Dye wastewater exhibits high color intensity, substantial organic content, and contains refractory synthetic dyes, intermediates, auxiliaries, and metal ions. It possesses toxicity, bioaccumulation potential, and photodegradation resistance, as well as being capable of long-term impacts on aquatic ecosystems and posing risks to human health through drinking water and food chains [4,5]. Developing efficient, economical, and environmentally friendly treatment technologies for dye wastewater has become an urgent priority. As an advanced oxidation process (AOP), photocatalytic technology can generate highly reactive species under light irradiation, achieving carbonization and mineralization of dye molecules into CO_2_ and inorganic salts [6]. Moreover, photocatalysis typically operates at room temperature and atmospheric pressure, leveraging solar energy to reduce energy consumption, and is regarded as a green, efficient approach for water treatment [7]. However, photocatalytic applications also face several limitations [8], including the following: common TiO_2_-based catalysts absorb ultraviolet light predominantly, exhibiting low utilization of visible light; photogenerated carriers tend to recombine, resulting in limited quantum yield; and there are stability issues with photocatalysts. Therefore, it is crucial to achieve further breakthroughs in the design and development of novel photocatalytic materials responsive to visible light.

Bi_2_O_2_CO_3_ (an n-type semiconductor) has emerged as a research hotspot in photocatalysis due to its environmental friendliness, economical cost, and unique optoelectronic properties [9,10]. Bi_2_O_2_CO_3_ exhibits a typical Aurivillius layered structure, with its crystal composed of alternately stacked (Bi_2_O_2_)^2+^ and (CO_3_)^2−^ layers [11,12,13,14]. Bi_2_O_2_CO_3_ possesses a relatively wide bandgap (approximately 3.5 eV), along with stable chemical properties and non-toxicity [15]. Its valence band is primarily composed of O 2p orbitals, exhibiting a high oxidation potential that enables the generation of highly reactive oxidizing radicals such as ·OH for the oxidative degradation of organic contaminants [16]. However, Bi_2_O_2_CO_3_ also exhibits limitations as a photocatalyst. On one hand, its wide bandgap permits only ultraviolet light absorption, resulting in a weak visible-light response [17]; on the other hand, the photogenerated electron–hole pairs easily undergo rapid recombination, leading to low quantum efficiency and hindering the full utilization of photocatalytic efficiency. In addition, Bi_2_O_2_CO_3_ possesses a limited number of active sites, further restricting its application effectiveness in actual pollutant degradation. Constructing a heterojunction represents an effective strategy to overcome these bottlenecks. Qiu et al. [18] synthesized the p-n heterojunction Bi_2_O_2_CO_3_/BiOBr via the solvothermal method for removing Rhodamine B (RhB) from water. The degradation efficiency of pure Bi_2_O_2_CO_3_ reached 10% under visible-light irradiation within 40 min, while the 1:8 Bi_2_O_2_CO_3_/BiOBr composite removed 94% of RhB within 20 min. Xue et al. [9] hydrothermally synthesized the heterostructure Bi_4_O_5_I_2_/Bi_2_O_2_CO_3_ photocatalyst. The optimal sample achieved a degradation efficiency of tetracycline hydrochloride (TC) up to 80.6% within 30 min of visible-light irradiation (significantly superior to each individual component). The reaction rate constants of the composite material were 2.4 times and 10.9 times those of Bi_4_O_5_I_2_ and Bi_2_O_2_CO_3_, respectively. The internal electric field within the heterojunction suppressed photogenerated carrier recombination effectively, thus enhancing photocatalytic degradation performance.

The heterojunction structure generates the internal electric field at the interface, efficiently driving the separation of photogenerated carriers while effectively prolonging their lifetime and enhancing their redox capabilities [19]. This electric field effect not only broadens the light absorption range but also suppresses carrier recombination, which significantly enhances photocatalytic activity and cycling stability. The n-type semiconductor AgVO_3_ is regarded as a promising photocatalyst for environmental remediation due to its narrow bandgap (E_g_ ~ 2.0–2.5 eV) and moderate band position, making it suitable for visible-light-induced redox processes [20,21]. One-dimensional nanoribbons/nanowires are commonly observed morphologies, facilitating directed carrier transport and enhanced light absorption, resulting in the outstanding performance in organic contaminant degradation [22]. However, AgVO_3_ also faces critical limitations. Under light irradiation, it readily undergoes rapid charge-carrier recombination, photocorrosion, and exhibits low quantum efficiency, which together further constrain its applicability in photocatalysis. Consequently, the construction of a heterojunction has been widely adopted to enhance the transport efficiency of photogenerated carriers through the internal electric field and band structure regulation at the interface. Liu et al. [23] constructed an S-type AgVO_3_/Ag_2_S heterojunction photocatalyst via the hydrothermal route. The degradation efficiencies of Rhodamine B and tetracycline hydrochloride reached 99% (25 min) and 72% (120 min), respectively, under visible-light excitation. Alzahrani and Ismail [24] prepared an n-n heterojunction AgVO_3_/WO_3_ for the removal of ciprofloxacin. The optimized 9% AgVO_3_/WO_3_ sample achieved near-complete removal of the pollutant within 120 min, with an apparent rate constant approximately 18-fold higher than that of WO_3_. Bavani et al. [25] synthesized a AgVO_3_/BiOI heterojunction photocatalyst by the hydrothermal method for Rhodamine B wastewater treatment, with the 1wt% AgVO_3_/BiOI exhibiting superior photocatalytic activity. The reaction rate constants for BiOI, AgVO_3_, and 1wt% AgVO_3_/BiOI were 0.006, 0.0033, and 0.0255 min^−1^, respectively.

Based on this, this work prepared a novel n-n heterojunction Bi_2_O_2_CO_3_/AgVO_3_ composite photocatalyst with visible-light responsiveness by in situ chemical deposition. Characterization technologies, including XRD, SEM, TEM, EDS, FT-IR, XPS, UV–Vis DRS, and PL, were employed to investigate the crystal phases, morphology and structure, composition and chemical information, light absorption properties, and photogenerated carrier recombination rates of the prepared samples. The photocatalytic performance and cycling stability of the synthesized nanocomposite were evaluated with contaminant degradation. The effects of typical reaction parameters (photocatalyst dosage and initial contaminant concentration) on the photocatalytic performance of Bi_2_O_2_CO_3_/AgVO_3_ were examined. Characterization results, radical trapping experiments, and UV–Vis absorption spectroscopy of photodegradation reaction solutions were combined to elucidate the photocatalytic degradation process and mechanism. The novel visible-light-driven photocatalyst prepared in this study exhibits outstanding photocatalytic performance and stability, offering a potentially efficient and sustainable solution for environmental remediation.

## 2. Experimental Section

### 2.1. Chemicals

Anhydrous sodium carbonate (Na_2_CO_3_) and methylene blue (MB) were purchased from Sinopharm Chemical Reagent Co., Ltd. (Shanghai, China) and Aladdin Biochemical Technology Co., Ltd. (Shanghai, China), respectively. Silver nitrate (AgNO_3_) and absolute ethanol were procured from Hubio (Shenzhen, China) and Meryer Biochemical Technology Co., Ltd. (Shanghai, China), respectively. Bismuth nitrate pentahydrate (Bi(NO_3_)_3_·5H_2_O, 99%), hexadecyl trimethyl ammonium bromide (CTAB, 99%), ammonium metavanadate (NH_4_VO_3_, 99% metals basis), triethanolamine (TEOA), tert-butanol (TBA, 99%), and p-benzoquinone (BQ, 99%) were supplied by Macklin Biochemical Technology Co., Ltd. (Shanghai, China). All reagents used for photocatalyst preparation and activity evaluation experiments were of analytical grade. Deionized water with a conductivity of 1 μS/cm was used throughout the experiments.

### 2.2. Preparation of Bi_2_O_2_CO_3_/AgVO_3_

Bi_2_O_2_CO_3_ with a nanosheet-like surface morphology was prepared by chemical deposition. A total of 0.50 g of CTAB and 2.99 g of Na_2_CO_3_ were dissolved in 100 mL of deionized water. The mixture was stirred magnetically until dissolved to obtain the transparent solution (labeled as Solution A). Next, 1.71 g of Bi(NO_3_)_3_·5H_2_O was dissolved in 30 mL of HNO_3_ (1 M) solution and sonicated until clear, marking this solution as Solution B. Solution B was added dropwise to the continuously stirred Solution A, accompanied by the generation of abundant foam and particles. The mixture was stirred at room temperature for 3 h. The suspension after the reaction was filtered and washed three times with deionized water and anhydrous ethanol, respectively. The resulting white precipitate was dried in a drying oven at 80 °C for 8 h, cooled to room temperature, and ground evenly to obtain the white fine powder of Bi_2_O_2_CO_3_.

Bi_2_O_2_CO_3_/AgVO_3_ heterojunction composite photocatalysts with Bi_2_O_2_CO_3_-to-AgVO_3_ molar ratios of 2:1, 3:1, 4:1, 5:1, and 6:1 were synthesized by the chemical in situ deposition method, and were labeled as BCAV2, BCAV3, BCAV4, BCAV5, and BCAV6, respectively. The preparation process is described using the BCAV4 sample as an example: Bi_2_O_2_CO_3_ (0.2 mmol) was dispersed in 30 mL of deionized water and ultrasonicated for 30 min. NH_4_VO_3_ (0.1 mmol) was dissolved in 30 mL of deionized water maintained at 80 °C. The NH_4_VO_3_ solution was slowly added to the continuously stirred Bi_2_O_2_CO_3_ suspension, followed by the slow dropwise addition of 20 mL of AgNO_3_ (0.005 M) under continuous stirring for 3 h. After filtration, the precipitate was washed three times with deionized water and three times with anhydrous ethanol, then dried in an oven at 60 °C for 12 h. After cooling to room temperature, the precipitate was ground uniformly to obtain the Bi_2_O_2_CO_3_/AgVO_3_ composite sample. The pure AgVO_3_ sample was prepared following the same method without the addition of Bi_2_O_2_CO_3_.

### 2.3. Material Characterizations

The crystal structures of Bi_2_O_2_CO_3_, AgVO_3_, and Bi_2_O_2_CO_3_/AgVO_3_ composite samples were determined by X-ray diffraction (XRD, SmartLab SE, Rigaku, Tokyo, Japan) under Cu-Kα radiation. Scanning electron microscopy (SEM, Sigma 360, ZEISS, Oberkochen, Germany) combined with energy-dispersive X-ray spectroscopy (EDS, Xplore 30, Oxford Instruments, Abingdon, UK) was used to investigate the morphology and elemental mapping of the photocatalysts. The microstructure and lattice information of the photocatalysts were determined by transmission electron microscopy (TEM, JEM-F200, JEOL, Yokohama, Japan) at a voltage of 200 kV. A Fourier-transform infrared spectrometer (FT-IR, Nicolet iS20, Thermo Fisher Scientific, Waltham, MA, USA) was employed to analyze the chemical composition and molecular structure of the materials. The elemental composition and chemical valence states of the samples were characterized by X-ray photoelectron spectroscopy (XPS, K-Alpha, Thermo Scientific, Waltham, MA, USA). UV–Vis diffuse reflectance spectroscopy (UV–Vis DRS) on the UV–Vis–NIR spectrophotometer (UV-3600i Plus, Shimadzu, Kyoto, Japan) was adopted to examine the light absorption properties of photocatalysts. A fluorescence spectrometer (PL, FLS1000, Edinburgh Instruments, Abingdon, UK) with an excitation wavelength setting of 370 nm was utilized to evaluate the separation and complexation properties of photogenerated carriers of the materials.

### 2.4. Photocatalytic Performance Evaluation

The photocatalytic activity of Bi_2_O_2_CO_3_, AgVO_3_, and Bi_2_O_2_CO_3_/AgVO_3_ was evaluated by the degradation of organic dye methylene blue (MB) in aqueous solution. A visible light source was provided by a 300 W xenon lamp (YM-GHX-XE-300, YuMing, Shanghai, China) equipped with the 400 nm cutoff filter. Degradation experiments were conducted in a double-jacketed photocatalytic glass reactor. Circulating cooling water was pumped into the reactor jacket to maintain the reaction system temperature at room temperature. Prior to irradiation, 50 mg of photocatalyst was suspended in 100 mL of MB (10 mg/L), and the mixture was magnetically stirred in the dark for 30 min to allow the adsorption–desorption equilibrium to be achieved. After the xenon lamp was activated, 4 mL of the sample was collected every 15 min and centrifuged at 10,000 rpm to separate the photocatalyst and MB solution. The absorbance of the supernatant was measured using the UV–visible spectrophotometer (L8, YOKE, Shanghai, China) at the characteristic absorption wavelength of 664 nm. The photocatalytic degradation efficiency and degradation reaction kinetics of MB were calculated based on Equations (1) and (2).

(1) $$\eta =\frac{C_{0}-C_{t}}{C_{0}}$$

(2) $$\ln(\frac{C_{0}}{C_{t}})=kt$$

where *η* is the degradation efficiency, %; *C*_0_ is the initial MB concentration before the photocatalytic reaction, mg/L; *C_t_* is the solution concentration in the reactor at *t* min of illumination, mg/L; *k* is the kinetic constant, min^−1^; *t* is the illumination time, min.

## 3. Results and Discussion

### 3.1. XRD Analysis

**Figure 1:**
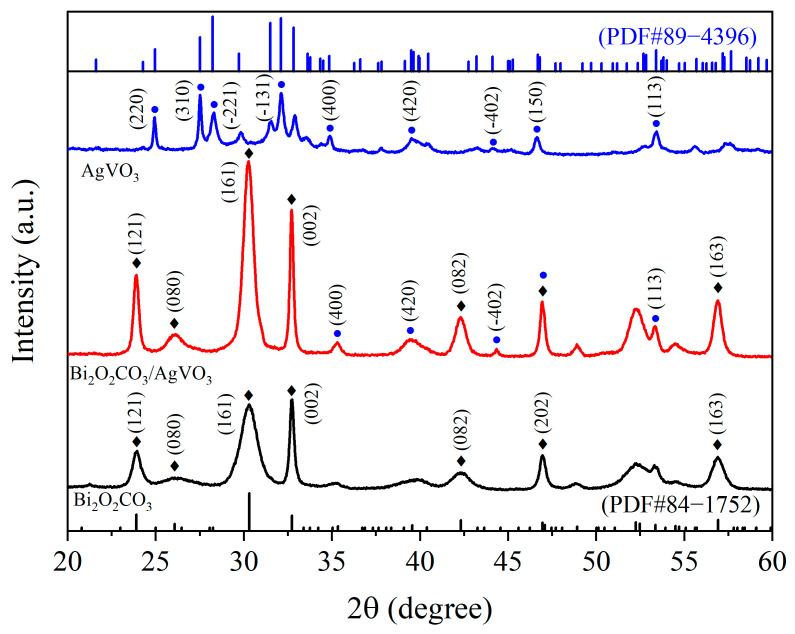
XRD patterns of Bi_2_O_2_CO_3_, AgVO_3_, and Bi_2_O_2_CO_3_/AgVO_3_.

To analyze the crystalline phases of the prepared samples, the XRD patterns of Bi_2_O_2_CO_3_, AgVO_3_, and Bi_2_O_2_CO_3_/AgVO_3_ are presented in Figure 1. As shown in the figure, the main characteristic diffraction peaks of the Bi_2_O_2_CO_3_ pattern at 23.90°, 26.07°, 30.30°, 32.73°, 42.31°, 46.96°, and 56.92° correspond to the (121), (080), (161), (002), (082), (202), and (163) diffraction crystal planes, respectively, consistent with the standard pattern (JCPDS No. 84-1752) of the tetragonal-phase Bi_2_O_2_CO_3_ [9]. The diffraction peaks of AgVO_3_ at 24.95°, 27.50°, 28.23°, 32.12°, 34.85°, 39.51°, 44.11°, 46.69°, and 53.40° correspond to the (220), (310), (−221), (−131), (400), (420), (−402), (150), and (113) diffraction planes, respectively, in good agreement with the monoclinic-phase AgVO_3_ (JCPDS No. 89-4396) [26,27]. The diffraction pattern of the Bi_2_O_2_CO_3_/AgVO_3_ composite sample reveals the main diffraction peaks of both Bi_2_O_2_CO_3_ and AgVO_3_, providing preliminary evidence for the successful synthesis of composite material. Furthermore, some characteristic diffraction peaks of AgVO_3_ are close to or overlap with those of Bi_2_O_2_CO_3_, such as the diffraction peak near 46.9°. The Bi_2_O_2_CO_3_/AgVO_3_ pattern exhibits sharp and distinct characteristic diffraction peaks without significant shift. In addition, no other impurity peaks appear, confirming the high crystallinity of the composite photocatalyst and indicating that the crystal structure of Bi_2_O_2_CO_3_ remained unchanged during synthesis.

### 3.2. SEM, EDS, and TEM Analyses

**Figure 2:**
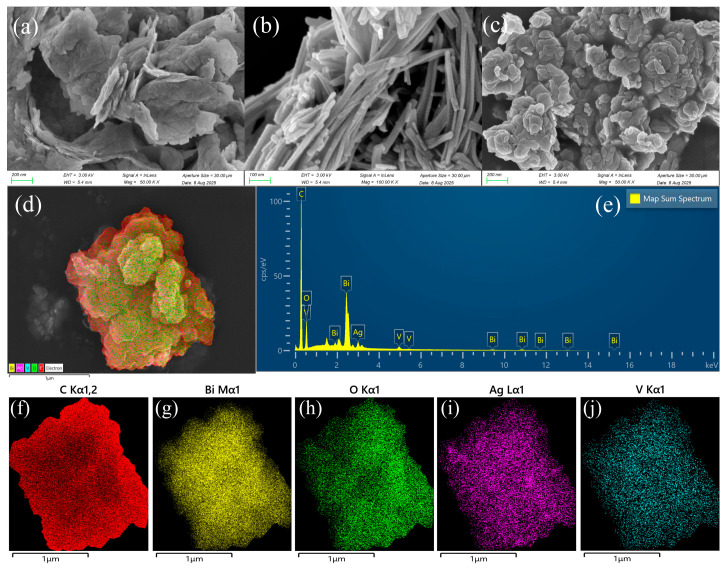
SEM images of Bi_2_O_2_CO_3_ (**a**), AgVO_3_ (**b**), and Bi_2_O_2_CO_3_/AgVO_3_ (**c**); EDS of BCAV4 (**d**,**e**); and SEM-EDS elemental mapping of BCAV4 (**f**–**j**).

Morphological and microstructural analyses of Bi_2_O_2_CO_3_, AgVO_3_, and Bi_2_O_2_CO_3_/AgVO_3_ nanomaterials were performed utilizing SEM and TEM. Figure 2a displays the morphology of Bi_2_O_2_CO_3_ as irregular flake-like aggregates composed of numerous nanosheets, with the dimensions of nanosheets generally within 200 nm. The synthesized AgVO_3_ exhibits more uniform nanorod morphology (Figure 2b), with the average diameter around 40 nm. Figure 2c shows that the Bi_2_O_2_CO_3_/AgVO_3_ nanocomposite exhibits the morphological characteristics of both components, with AgVO_3_ nanorods embedded in the surface of the Bi_2_O_2_CO_3_ semiconductor nanosheets. Obviously, the surface roughness of the nanocomposite has increased. The formation of a heterojunction between the components facilitates the adsorption of a large amount of MB pollutant while providing more active sites for photocatalytic degradation. In order to obtain compositional information of the prepared nanocomposite, EDS-mapping characterization was performed on BCAV4. Figure 2d–e reveal that BCAV4 consists of C, Bi, O, Ag, and V elements, providing visual evidence of the elemental distribution. More importantly, uniform distribution of the C, Bi, O, Ag, and V elements was observed on the surface of BCAV4 (Figure 2f–j). This suggests that AgVO_3_ nanorods are uniformly loaded onto the surface of Bi_2_O_2_CO_3_ nanosheets, which confirms the successful preparation of the Bi_2_O_2_CO_3_/AgVO_3_ nanocomposite.

**Figure 3:**
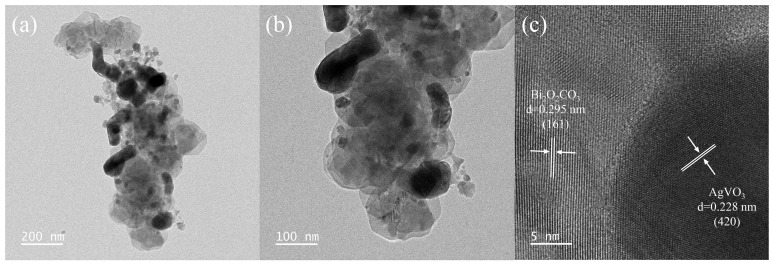
TEM images (**a**,**b**) and HR-TEM image (**c**) of BCAV4.

Figure 3 illustrates the TEM analysis of the BCAV4 nanocomposite. Figure 3a shows that the microstructure of Bi_2_O_2_CO_3_ exhibits a regular nanosheet-like structure, with black AgVO_3_ nanorods densely loaded on the nanosheet surface. This is consistent with the SEM characterization results. Figure 3b clearly reveals the tight interfacial bonding between the Bi_2_O_2_CO_3_ and AgVO_3_ components. HR-TEM clearly reveals the lattice fringes of the BCAV4 composite photocatalyst (Figure 3c). The measured lattice spacings of 0.295 nm and 0.228 nm are assigned to the (161) plane of Bi_2_O_2_CO_3_ and the (420) plane of AgVO_3_, respectively, which confirms heterojunction formation at the interface and is consistent with the XRD analysis. The formation of BCAV4 heterojunction facilitates the migration and separation of photogenerated carriers, thereby enhancing photocatalytic activity.

### 3.3. FT-IR Characterization

**Figure 4:**
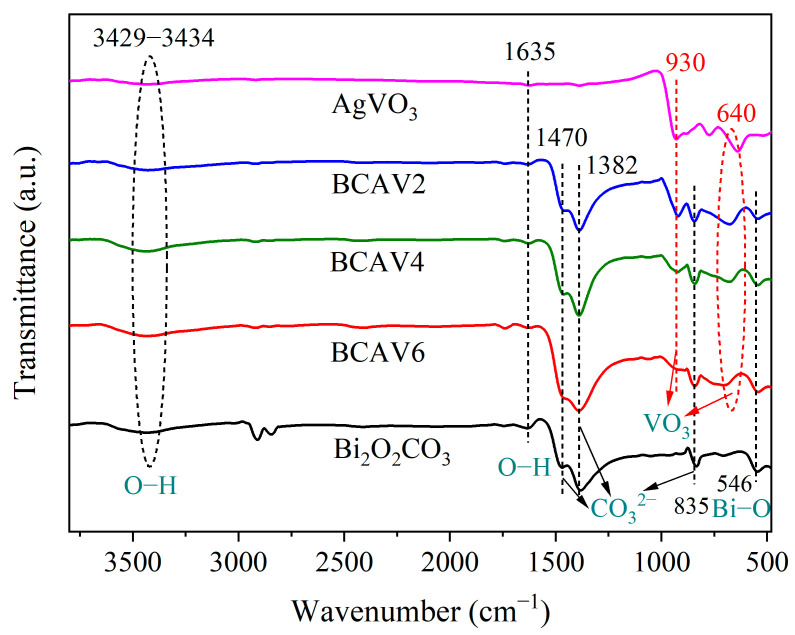
FT-IR spectra of Bi_2_O_2_CO_3_, AgVO_3_, and Bi_2_O_2_CO_3_/AgVO_3_.

FT-IR was used to analyze the chemical bonds and functional groups of Bi_2_O_2_CO_3_, AgVO_3_, and Bi_2_O_2_CO_3_/AgVO_3_ nanocomposites with different loading ratios. As shown in Figure 4, the infrared spectra of BCAV2, BCAV4, and BCAV6 exhibit all the characteristic peaks of Bi_2_O_2_CO_3_ and AgVO_3_, with no additional impurity signals detected. The characteristic peaks at 3429–3434 cm^−1^ and 1635 cm^−1^ are assigned to the O–H stretching and bending vibrations of surface-adsorbed H_2_O molecules, respectively [28]. The absorption peaks at 640 cm^−1^ and 930 cm^−1^ in the AgVO_3_ and Bi_2_O_2_CO_3_/AgVO_3_ spectra are assigned to the symmetric stretching of the VO_3_ group [29]. The intensity of the absorption peaks gradually increases with increasing Ag content. The absorption peak observed at 546 cm^−1^ in the spectra of Bi_2_O_2_CO_3_ and Bi_2_O_2_CO_3_/AgVO_3_ nanocomposites is due to the stretching vibration mode of the Bi–O bond [30]. Furthermore, the absorption peak at 835 cm^−1^ belongs to the out-of-plane bending vibration of the CO_3_^2−^ group, and the characteristic peaks at 1382 cm^−1^ and 1470 cm^−1^ originate from the antisymmetric stretching vibration of CO_3_^2−^ [31,32]. The infrared spectrum provides direct evidence for the coexistence of Bi_2_O_2_CO_3_ and AgVO_3_ in the composite sample.

### 3.4. XPS Analysis

**Figure 5:**
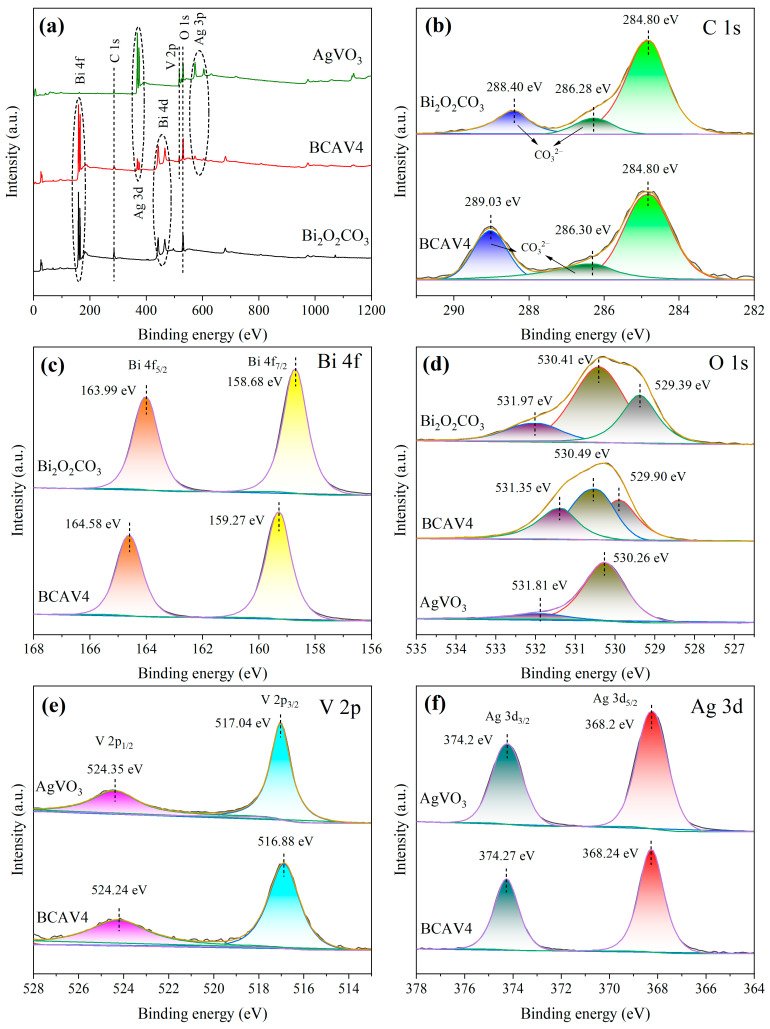
XPS full spectra of Bi_2_O_2_CO_3_, AgVO_3_, and BCAV4 (**a**); high-resolution scanning spectra of C 1s orbitals (**b**) and Bi 4f orbitals (**c**) in Bi_2_O_2_CO_3_ and BCAV4; O 1s orbitals (**d**) in Bi_2_O_2_CO_3_, AgVO_3_, and BCAV4; V 2p orbitals (**e**) and Ag 3d orbitals (**f**) in AgVO_3_ and BCAV4.

To reveal the elemental composition and chemical state of the synthesized samples, Bi_2_O_2_CO_3_, AgVO_3_, and BCAV4 were characterized by XPS, and the results are shown in Figure 5. All elements were calibrated based on the C l s standard peak (binding energy = 284.80 eV). The XPS full spectrum (Figure 5a) reveals characteristic peaks attributable to Ag, V, and O elements within the AgVO_3_ spectrum, while the Bi_2_O_2_CO_3_ spectrum contains characteristic peaks belonging to Bi, C, and O elements. Meanwhile, the BCAV4 spectrum exhibits photoelectron peaks for all these elements, consistent with the EDS-mapping analysis results. From the C ls spectra of Bi_2_O_2_CO_3_ and BCAV4 samples shown in Figure 5b, it can be seen that the characteristic peak at the binding energy of 284.80 eV belongs to adventitious carbon (used for correcting photoelectron peaks). The characteristic peaks at 286.28 eV and 288.40 eV in the Bi_2_O_2_CO_3_ spectrum are attributed to the CO_3_^2−^ group [33], while in the BCAV4 spectrum, these peaks shift toward 286.30 eV and 289.03 eV, respectively. This confirms the presence of CO_3_^2−^ in BCAV4.

The high-resolution Bi 4f scan patterns of Bi_2_O_2_CO_3_ and BCAV4 samples are presented in Figure 5c. Peaks at 158.68 eV and 163.99 eV in the Bi_2_O_2_CO_3_ XPS spectrum are characteristic of the Bi 4f_7/2_ and Bi 4f_5/2_ binding energies, respectively. The two diffraction peaks in the BCAV4 spectrum shift toward 159.27 eV and 164.58 eV, respectively, with a common shift of 0.59 eV. This confirms the presence of Bi^3+^ in both Bi_2_O_2_CO_3_ and BCAV4 samples. Furthermore, the increased binding energy of characteristic peaks in composite nanomaterials demonstrates the occurrence of chemical interactions at the interface between Bi_2_O_2_CO_3_ and AgVO_3_.

Figure 5d depicts the O 1s spectra of Bi_2_O_2_CO_3_, AgVO_3_, and BCAV4 samples. The characteristic peaks at 529.39 eV and 530.26 eV in the Bi_2_O_2_CO_3_ and AgVO_3_ spectra are attributed to lattice oxygen. Additionally, the characteristic peaks at 531.97 eV and 531.81 eV are assigned to surface-adsorbed hydroxyl oxygen [34]. The characteristic peaks in the BCAV4 composite material shift to 529.90 eV and 531.35 eV, respectively. More importantly, the characteristic peak at 530.41 eV in the Bi_2_O_2_CO_3_ spectrum and the peak at 530.49 eV in the BCAV4 spectrum both correspond to CO_3_^2−^ [35].

The V 2p spectrum (Figure 5e) reveals characteristic peaks at 517.04 eV and 524.35 eV in the AgVO_3_ spectrum that correspond to the V 2p_3/2_ and V 2p_1/2_ orbitals of the V^5+^ ion, respectively. The characteristic peaks in the BCAV4 spectrum shift toward 516.88 eV and 524.24 eV, with corresponding binding energy shifts of 0.16 eV and 0.11 eV. The decrease in characteristic peak binding energy of the nanocomposite indicates enhanced chemical bonding interactions. The scanning spectrum of the Ag 3d orbital (Figure 5f) reveals two spin–orbit split peaks at 368.2 eV and 374.2 eV within the AgVO_3_ spectrum, attributed to the Ag 3d_5/2_ and Ag 3d_3/2_ orbitals of the Ag^+^ ion, respectively. In addition, the corresponding diffraction peaks in the BCAV4 spectrum shift to binding energies of 368.24 eV and 374.27 eV, respectively. This supports the presence of V^5+^ and Ag^+^ ions in the AgVO_3_ and BCAV4 samples.

Based on the above characterization results, it can be observed that the binding energies of the C ls, Bi 4f, O 1s, V 2p, and Ag 3d orbitals in the BCAV4 nanocomposite are shifted to a certain extent compared to those of the individual Bi_2_O_2_CO_3_ and AgVO_3_ nanocomposites. This demonstrates that the interface between the two components of the composite photocatalyst is in close contact and chemically interacts, which is consistent with the FT-IR analysis results. It further elucidates that the heterojunction has successfully formed within the nanocomposite BCAV4, effectively suppressing the rapid recombination of photogenerated carriers.

### 3.5. Optical Properties

**Figure 6:**
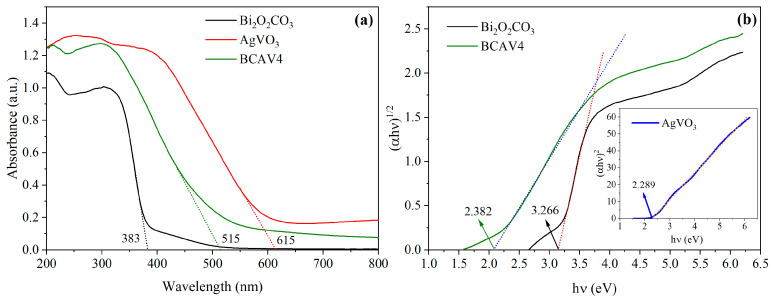
UV–Vis DRS (**a**) and corresponding band gap widths (**b**) of Bi_2_O_2_CO_3_, AgVO_3_, and BCAV4.

The light absorption properties and bandgap widths of Bi_2_O_2_CO_3_, AgVO_3_, and BCAV4 were investigated using UV–Vis DRS, with the results shown in Figure 6. Figure 6a reveals that the light absorption thresholds of Bi_2_O_2_CO_3_, AgVO_3_, and the nanocomposite reach 383 nm, 615 nm, and 515 nm, respectively. Clearly, the single Bi_2_O_2_CO_3_ component exhibits negligible absorption in the visible spectrum, with its primary light-responsive region confined to ultraviolet light. In contrast, the AgVO_3_ sample demonstrates excellent light absorption properties in the visible region. Compared to pure Bi_2_O_2_CO_3_, the BCAV4 composite sample formed through the heterojunction exhibits a significant red shift in its light absorption range toward the visible spectrum, essentially covering the entire visible wavelength band. In addition, the enhanced light absorption intensity of the BCAV4 nanocomposite effectively improves its visible light response and conversion capabilities, thus enhancing the yield of photogenerated electron–hole pairs.

The bandgap value (E_g_) of photocatalyst samples can be calculated using the Tauc equation [36], as follows:

(3) $${\left(\alpha hv\right)}^{n}=A(hv-E_{g})$$

where *α*, *h*, and *ν* represent the absorption coefficient, Planck’s constant, and optical frequency, respectively; n depends on the transition type of semiconductor; A is a material-dependent constant; E_g_ is the bandgap width, eV; Bi_2_O_2_CO_3_ is the indirect bandgap semiconductor (n = 1/2); AgVO_3_ is the direct bandgap semiconductor (n = 2).

The bandgap widths of Bi_2_O_2_CO_3_, AgVO_3_, and BCAV4 were estimated based on Equation (3) (Figure 6b). The bandgap energies of Bi_2_O_2_CO_3_, AgVO_3_, and the nanocomposite are 3.266 eV, 2.289 eV, and 2.382 eV, respectively. Compared to the single-component Bi_2_O_2_CO_3_, the bandgap width of composite material decreased by 0.884 eV. This suggests that the energy required for photon excitation is significantly minimized after the two components form a heterojunction, which facilitates the generation of electron–hole pairs under visible light and, consequently, enhances photocatalytic activity.

### 3.6. Photoluminescence Analysis

**Figure 7:**
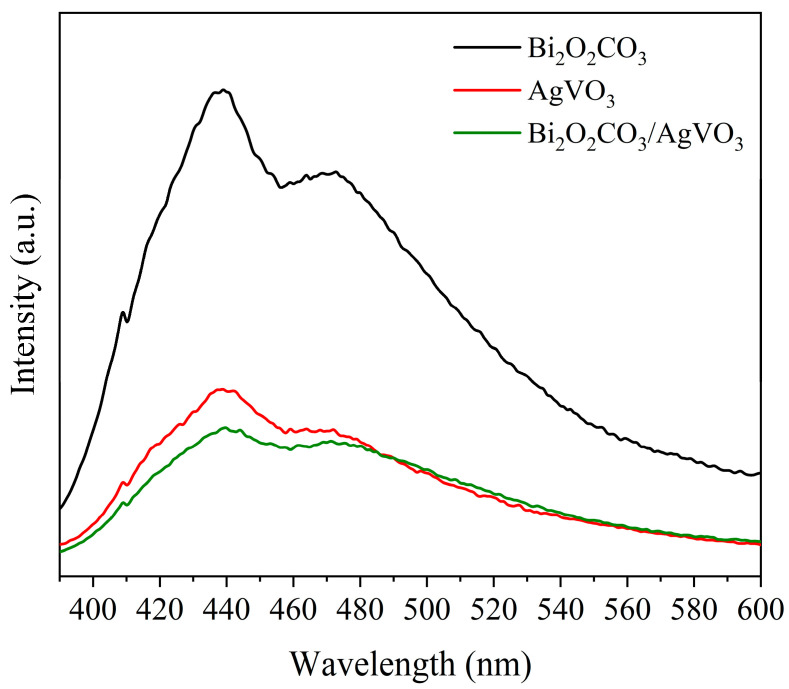
Photoluminescence spectra of Bi_2_O_2_CO_3_, AgVO_3_, and BCAV4.

Photoluminescence spectra characterize the fluorescence emission intensity of photocatalyst samples, serving to evaluate the charge transfer efficiency of photogenerated electron–hole pairs. Generally, a lower fluorescence emission intensity indicates higher separation efficiency of the photoinduced carriers [37]. Figure 7 presents the photoluminescence spectra of Bi_2_O_2_CO_3_, AgVO_3_, and BCAV4. All three materials exhibit fluorescence emission peaks near the wavelength of 440 nm. Bi_2_O_2_CO_3_ displays the highest fluorescence intensity, reflecting a relatively high rate of electron–hole recombination under light excitation. The fluorescence intensity of the Bi_2_O_2_CO_3_/AgVO_3_ nanocomposite is the lowest, slightly less than the emission peak intensity of AgVO_3_. Formation of the heterojunction reduces the recombination rate of photoinduced charge carriers, which substantially lowers the fluorescence intensity and enhances the photocatalytic activity compared with the individual components.

### 3.7. Photocatalytic Degradation Performance

**Figure 8:**
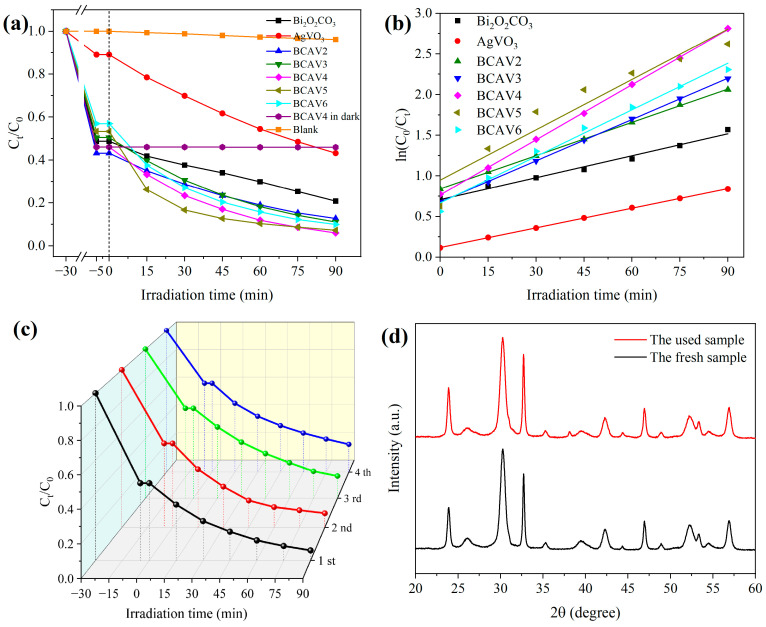
Photocatalytic degradation efficiency (**a**) and degradation kinetic curves (**b**) of MB by Bi_2_O_2_CO_3_, AgVO_3_, and Bi_2_O_2_CO_3_/AgVO_3_; cycling degradation performance of BCAV4 on MB (**c**); and XRD patterns of BCAV4 before and after cycling experiments (**d**).

The photocatalytic degradation activity of Bi_2_O_2_CO_3_, AgVO_3_, and Bi_2_O_2_CO_3_/AgVO_3_ nanocomposites with different molar ratios was evaluated based on the degradation efficiency of the target pollutant MB solution, with results displayed in Figure 8a. The blank experiment showed that after 90 min of irradiation (without any photocatalyst), the degradation efficiency of MB due to self-photolysis was only 3.86%. It was observed that the AgVO_3_ component removed only 56.77% of MB after 90 min of photocatalytic degradation, with an adsorption rate of 10.93% when kept in the dark for 30 min. This is attributed to the easy recombination of photoexcited electron–hole pairs, resulting in decreased photocatalytic activity. In addition, the Bi_2_O_2_CO_3_ component demonstrated a degradation efficiency of 79.15% for MB after 90 min of visible-light irradiation, attributed to its superior adsorption capacity. Under dark conditions, the adsorption rate of MB reached 51.27% as it approached adsorption equilibrium. This is because Bi_2_O_2_CO_3_ possesses a larger specific surface area, enabling it to provide more adsorption sites compared to AgVO_3_. After the two components were combined to form the heterojunction, the MB degradation efficiency significantly improved following 90 min of visible-light irradiation. This indicates that the photocatalytic activity of the Bi_2_O_2_CO_3_/AgVO_3_ nanocomposite was enhanced, along with enhanced transport efficiency of photogenerated charge carriers. After 90 min of photocatalytic reaction, the degradation efficiencies of BCAV2, BCAV3, BCAV4, BCAV5, and BCAV6 for MB were 87.27%, 88.88%, 94.00%, 92.74%, and 90.04%, respectively. The different molar ratios of components significantly influenced the photocatalytic performance of nanocomposites. Evidently, BCAV4 exhibited the highest photocatalytic activity. Following adsorption equilibrium, the BCAV4 nanocomposite was stirred for 90 min in the absence of light to serve as a control for verifying visible-light-induced photocatalytic activity. The degradation of MB showed almost no change upon reaction completion.

Figure 8b exhibits the degradation kinetic curves of MB for the Bi_2_O_2_CO_3_, AgVO_3_, and Bi_2_O_2_CO_3_/AgVO_3_ samples. Fitting results indicate that the photocatalytic degradation of MB follows pseudo-first-order reaction kinetics. Table 1 presents the corresponding reaction kinetic models and detailed parameters for the photocatalytic degradation of MB. As shown in the table, BCAV4 in the Bi_2_O_2_CO_3_/AgVO_3_ composite sample exhibits the fastest photocatalytic degradation reaction rate (k = 0.0226 min^−1^), which is 2.51 and 2.79 times faster than that of Bi_2_O_2_CO_3_ and AgVO_3_, respectively. The significant enhancement in MB degradation rate by the composite nanomaterial is attributed to the formation of the heterojunction, which inhibits the rapid recombination of photogenerated carriers efficiently.

The stability of the photocatalytic activity of Bi_2_O_2_CO_3_/AgVO_3_ nanocomposites is presented in Figure 8c. The degradation efficiency of MB decreased progressively from 94.00% to 80.31% as the number of degradation cycles increased. After four degradation cycles, the BCAV4 sample still removed over 80% of MB contaminants, demonstrating that the synthesized nanocomposite exhibits acceptable stability for reuse. XRD characterization was performed on BCAV4 samples before photodegradation and after four cycles of reuse (Figure 8d). It can be observed that the characteristic diffraction peaks of the photocatalyst did not exhibit significant shifts after cyclic degradation, and the peak shapes remained sharp with stable diffraction intensities, indicating that the crystalline phase remained stable. Furthermore, the weak diffraction peak near 38° in the cycled samples was attributed to residual impurities on the photocatalyst surface. In summary, the Bi_2_O_2_CO_3_/AgVO_3_ heterojunction nanocomposite exhibits excellent cyclic stability of photocatalytic activity.

### 3.8. Effect of Reaction Parameters on the Photocatalytic Activity of Bi_2_O_2_CO_3_/AgVO_3_

#### 3.8.1. Photocatalyst Dosage

**Figure 9:**
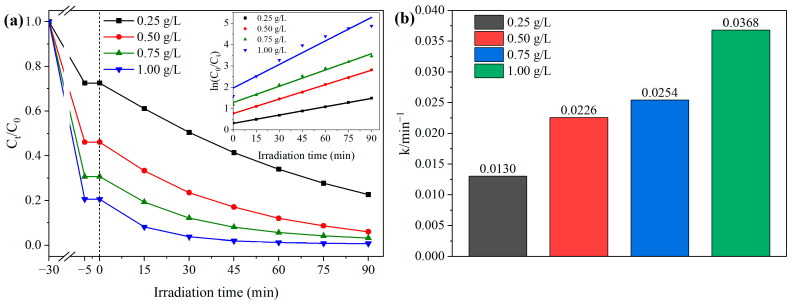
Effect of BCAV4 photocatalyst dosage on degradation efficiency, kinetic curve (**a**) and kinetic constant (**b**) of MB. C_MB_: 10mg/L.

The photocatalyst dosage affects the degradation capacity of the reaction system toward contaminants, with relevant results shown in Figure 9. As depicted in Figure 9a, when the system achieved adsorption equilibrium, the adsorption rate of BCAV4 for MB gradually increased with rising dosage. The adsorption performance of BCAV4 improved with increasing dosage, as follows: 27.47% (0.25 g/L), 53.92% (0.50 g/L), 69.31% (0.75 g/L), and 79.51% (1.00 g/L). This is attributed to the increased amount of photocatalyst providing more adsorption sites for MB. Following 90 min of visible-light exposure, the photocatalyst dosages of 0.25–1.00 g/L achieved MB degradation efficiencies of 77.40%, 94.00%, 96.85%, and 99.24%, respectively. Evidently, the photocatalytic degradation efficiency of BCAV4 for MB progressively improved with increasing dosage. At the dosage of 1.00 g/L, the photodegradation reaction can almost completely decompose all MB contaminants in water. This is due to the fact that increasing the photocatalyst dosage within a certain range could provide a large amount of MB adsorption sites and reactive sites, thereby generating more active components to act on the target pollutants. However, an excessive dosage will seriously reduce the light transmittance of the reaction system, significantly decreasing the number of photons absorbed by the photocatalyst and thereby diminishing photocatalytic activity. In summary, the optimal dosage of BCAV4 is selected as 0.50 g/L.

The inset in Figure 9a illustrates the photocatalytic degradation kinetic curves of MB under different BCAV4 dosages. The fitting results indicate that the photocatalytic degradation of MB follows the pseudo-first-order kinetic model at various photocatalyst dosages. Figure 9b presents the degradation kinetic constants corresponding to different photocatalyst dosages. Obviously, the photodegradation rate of MB gradually enhanced with increasing BCAV4 dosage. When the BCAV4 dosage was 0.25, 0.50, 0.75, and 1.00 g/L, the reaction kinetic constants reached 0.0130, 0.0226, 0.0254, and 0.0368 min^−1^, respectively. This is because the increased amount of photocatalyst in the solution could enhance the yield of active species, thus accelerating the decomposition of MB pollutant molecules.

#### 3.8.2. Initial Concentration of Contaminants

**Figure 10:**
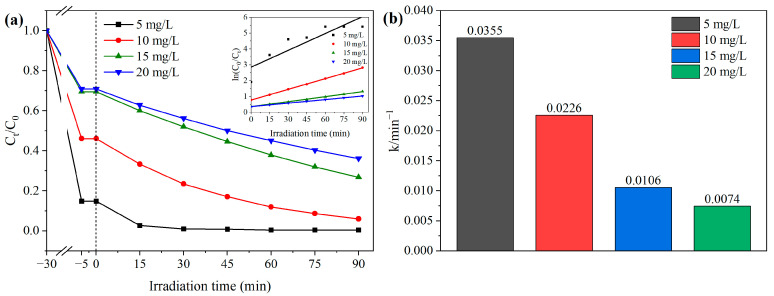
Effect of initial MB concentration on degradation efficiency, kinetic curve (**a**) and kinetic constant (**b**) of MB photoinduced by BCAV4. BCAV4 dosage: 0.50 g/L.

The influence of initial MB concentration on BCAV4 photocatalytic performance is presented in Figure 10. Figure 10a demonstrates that as the initial MB concentration increased, the adsorption rate of BCAV4 at equilibrium gradually decreased, with particularly poor adsorption performance at high initial concentrations. When the initial MB concentration was 15 and 20 mg/L, the adsorption rates of BCAV4 were 30.54% and 29.18%, respectively. Furthermore, the photocatalytic removal of MB by BCAV4 showed a gradual decline in efficiency with increasing initial concentration. When the initial MB concentrations were 5, 10, 15, and 20 mg/L, the photocatalytic degradation efficiencies reached 99.55%, 94.00%, 73.20%, and 63.86%, respectively. The inset in Figure 10a shows the photocatalytic degradation kinetic curves corresponding to various initial MB concentrations. Kinetic fitting shows that MB photodegradation under the tested initial concentrations obeys pseudo-first-order reaction kinetics.

Figure 10b illustrates a progressive reduction in the photocatalytic reaction rate of BCAV4 as the initial MB concentration increased, likely due to reduced light penetration at higher dye concentrations. The degradation kinetic constants corresponding to initial MB concentrations of 5–20 mg/L were 0.0355, 0.0226, 0.0106, and 0.0074 min^−1^, respectively. The increase in initial MB concentration caused a synchronous decrease in photocatalytic degradation efficiency and degradation reaction rate. This is due to the fact that, at a fixed photocatalyst dosage, the yield of active species per unit time remains relatively stable. As the number of MB molecules in water continued to increase, the active components could not degrade all the MB pollutant molecules, leading to inhibition of the photocatalytic reaction.

### 3.9. Photocatalytic Degradation Mechanism

**Figure 11:**
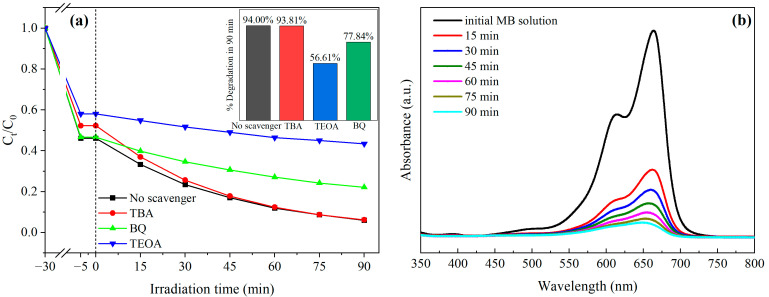
Scavenger study of MB photocatalytic degradation on Bi_2_O_2_CO_3_/AgVO_3_ photocatalyst (**a**); and UV–Vis absorption spectra of MB solution during visible-light photocatalytic degradation (**b**).

TBA, TEOA, and BQ were employed as scavengers to selectively quench ·OH, h^+^, and ·O_2_^−^, respectively, in order to elucidate the roles of reactive species in the Bi_2_O_2_CO_3_/AgVO_3_ photocatalytic degradation of MB. The results are presented in Figure 11a, and the inset of Figure 11a depicts the MB degradation efficiencies corresponding to different scavengers after 90 min of visible-light catalysis. Apparently, the addition of TBA reduced the MB degradation efficiency from 94.00% to 93.81%, a decrease of only 0.19%. This suggests that ·OH hardly participated in the photocatalytic degradation of MB by Bi_2_O_2_CO_3_/AgVO_3_. However, after adding TEOA and BQ to the solution, the photocatalytic degradation efficiency of Bi_2_O_2_CO_3_/AgVO_3_ for MB decreased to 56.61% and 77.84%, respectively, representing reductions of 37.39% and 16.16%. The significant inhibition of MB degradation upon the introduction of TEOA and BQ confirms that h^+^ and ·O_2_^−^ predominantly govern the photocatalytic process on Bi_2_O_2_CO_3_/AgVO_3_. The active species h^+^ and ·O_2_^−^ generated by the heterojunction effectively decomposed and disrupted the molecular structure of MB, resulting in the decolorization and degradation of MB contaminants.

The UV–Vis absorption spectrum of the MB solution during the visible-light photocatalytic reaction (Figure 11b) strongly confirms the effective decomposition of MB molecular structure. The MB solution was scanned over the wavelength range of 350–800 nm at intervals of 15 min. The absorbance of the maximum absorption peak at 664 nm gradually decreased with prolonged photodegradation time, and the characteristic absorption peak nearly disappeared after 90 min of reaction. This demonstrates that the active species, h^+^ and ·O_2_^−^, generated by Bi_2_O_2_CO_3_/AgVO_3_ under photoexcitation could efficiently destroy and decompose the chromophore group (phenothiazine conjugated chromophore) of MB molecules [38,39], thereby achieving effective photocatalytic degradation of MB contaminants in water.

**Figure 12:**
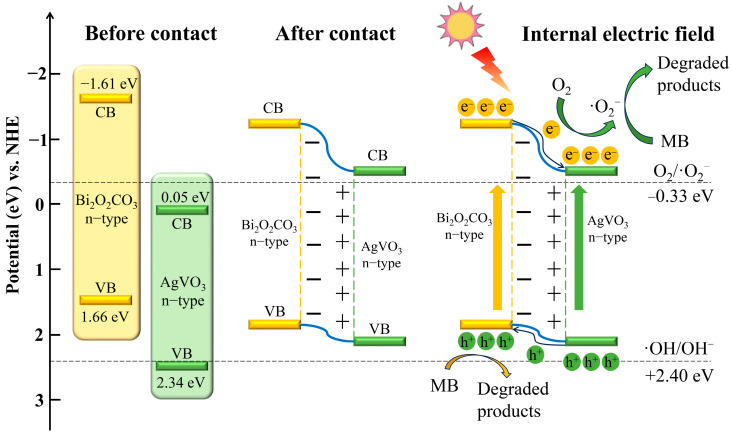
Possible photocatalytic mechanism of Bi_2_O_2_CO_3_/AgVO_3_ heterojunction towards MB.

Based on the aforementioned material characterization and radical scavenging analysis results, the potential photocatalytic degradation mechanism of MB by the n-n type Bi_2_O_2_CO_3_/AgVO_3_ heterojunction nanocomposite is proposed (Figure 12). Upon coupling the n-type semiconductors Bi_2_O_2_CO_3_ and AgVO_3_, a type-II heterojunction is established, facilitating effective bandgap alignment [40]. Electron transfer driven by the concentration gradient at the phase interface generates an internal electric field. Under visible-light excitation, Bi_2_O_2_CO_3_ and AgVO_3_ each generate photogenerated electron–hole pairs. The internal electric field facilitates charge migration, driving electrons from the conduction band of Bi_2_O_2_CO_3_ to that of AgVO_3_, and holes from the valence band of AgVO_3_ to that of Bi_2_O_2_CO_3_. This migration mechanism not only suppresses the recombination of photogenerated electron–hole pairs but also vigorously promotes the separation process of photogenerated carriers, thereby achieving photocatalytic activity superior to that of single-component systems. The increased binding energy of the Bi 4f characteristic peak and the decreased binding energy of the V 2p characteristic peak observed in the composite nanomaterial spectrum obtained from XPS characterization also serve as strong evidence.

Equations (4)–(7) represent the photodegradation reaction mechanisms. Upon visible-light irradiation, electrons accumulated in the AgVO_3_ conduction band capture O_2_ to undergo the reduction reaction, generating the strong oxidizing agent ·O_2_^−^. ·O_2_^−^ could oxidize and decompose MB to generate small-molecule degradation products. The strongly oxidizing h^+^, aggregated in the valence band of Bi_2_O_2_CO_3_, can directly capture and oxidatively remove MB molecules, resulting in CO_2_ and H_2_O as final products. Overall, the photocatalytic degradation of MB by Bi_2_O_2_CO_3_/AgVO_3_ is primarily governed by h^+^ and ·O_2_^−^ radicals. This finding is consistent with the active species capture results.

(4) $${\mathrm{Bi}}_{2}O_{2}{\mathrm{CO}}_{3}{/\mathrm{AgVO}}_{3}+hv\to {h_{\mathrm{VB}}}^{+}+{e_{\mathrm{CB}}}^{-}$$

(5) $${h_{\mathrm{VB}}}^{+}+{e_{\mathrm{CB}}}^{-}\to \mathrm{heat}$$

(6) $${e_{\mathrm{CB}}}^{-}+O_{2}\to \;\cdot {O_{2}}^{-}$$

(7) $$\mathrm{MB}+h^{+}/\cdot {O_{2}}^{-}\to {\mathrm{CO}}_{2}+H_{2}O+\mathrm{small}\;\mathrm{molecule}\;\mathrm{byproducts}$$

## 4. Conclusions

In summary, this research successfully synthesized a Bi_2_O_2_CO_3_/AgVO_3_ n-n heterojunction nanocomposite by the chemical deposition method. Characterization results reveal that the composite photocatalyst simultaneously contained characteristic diffraction peaks of both Bi_2_O_2_CO_3_ and AgVO_3_. AgVO_3_ nanorods were densely loaded onto the surface of Bi_2_O_2_CO_3_ nanosheets, with a uniform distribution of C, Bi, O, Ag, and V elements. XPS analysis provides direct evidence for the composition of the heterojunction. UV–Vis DRS and PL results indicate enhanced visible-light responsiveness in the Bi_2_O_2_CO_3_/AgVO_3_ heterojunction photocatalyst, accompanied by a decrease in bandgap energy. The photogenerated electron–hole pair separation efficiency of the nanocomposite improved significantly. MB degradation experiments demonstrate that BCAV4 nanocomposites exhibited outstanding photocatalytic activity and reusability. After 90 min of visible-light irradiation, BCAV4 achieved the maximum MB degradation efficiency of 99.55%, with photodegradation rates 2.51 and 2.79 times higher than those of Bi_2_O_2_CO_3_ and AgVO_3_, respectively. The improved photocatalytic activity can be ascribed to the effective construction of the n-n heterojunction. The electron transfer induced by the electron concentration gradient at the two-component interface caused the formation of an internal electric field, significantly enhancing the separation efficiency of photogenerated carriers. Moreover, the visible-light absorption capability was markedly improved. As the photocatalyst dosage increased and the initial MB concentration decreased, the pollutant degradation efficiency improved progressively. UV–Vis absorption spectra of the MB reaction solution and radical trapping revealed that the active species, h^+^ and ·O_2_^−^, generated under visible-light excitation could effectively disrupt and decompose the chromophore groups of MB molecules, thereby achieving efficient photodegradation of MB pollutants in water. This study lays the foundation for developing novel visible-light-driven photocatalytic materials and advancing their application in energy and environmental fields.

## Figures and Tables

**Table 1:** Photocatalytic degradation kinetic parameters of MB by Bi_2_O_2_CO_3_, AgVO_3_, and Bi_2_O_2_CO_3_/AgVO_3_.

Photocatalyst	Pseudo-First-Order Kinetic Model and Parameters		
	Kinetic Equations	Kinetic Constants (min^−1^)	R^2^
Bi_2_O_2_CO_3_	y = 0.7081 + 0.0090x	0.0090	0.9853
AgVO_3_	y = 0.1190 + 0.0081x	0.0081	0.9998
BCAV2	y = 0.8406 + 0.0136x	0.0136	0.9998
BCAV3	y = 0.6782 + 0.0169x	0.0169	0.9999
BCAV4	y = 0.7664 + 0.0226x	0.0226	0.9997
BCAV5	y = 0.9484 + 0.0206x	0.0206	0.9039
BCAV6	y = 0.6676 + 0.0191x	0.0191	0.9861

## Data Availability

The original contributions presented in this study are included in the article. Further inquiries can be directed to the corresponding author.
